# Whole genome sequencing of a wild swan goose population

**DOI:** 10.3389/fgene.2023.1038606

**Published:** 2023-02-24

**Authors:** Hongyu Ni, Yonghong Zhang, Yuwei Yang, Yijing Yin, Hengli Xie, Jinlei Zheng, Liping Dong, Jizhe Diao, Meng Wei, Zhichao Lv, Shouqing Yan, Yumei Li, Hao Sun, Xueqi Sun

**Affiliations:** ^1^ College of Animal Science, Jilin University, Changchun, China; ^2^ Jilin Province Economic Management Cadre College, Changchun, China; ^3^ Jilin Academy of Agricultural Sciences, Changchun, China

**Keywords:** swan goose, whole genome sequencing (WGS), SNP, linkage disequilibrium, genetic resource

## Introduction

It was reported that Chinese goose breeds are thought to have originated from wild swan geese (*Anser cygnoides*) (Shi et al., 2006). The wild swan goose, as a migratory waterfowl, has many different characteristics compared to the domestic goose breeds. Most notable is that the wild swan goose has long-distance flight ability. At present, the swan goose has two biological migration routes, namely inland migration routes and coastal migration routes. The majority of swan goose individuals in northeastern Mongolia (Inner Mongolia, Heilongjiang, and Jilin province) relocated to the Chinese Yangtze River, and the Russian Far East relocated to the coast of southeast China for winter (Zhu et al., 2020). Due to the lack of analysis of the genomic characteristics and selection signals of wild swan geese, the genetic basis underlying these characteristics is still not well investigated.

The swan goose has been listed as vulnerable in the Red List of Threatened Species of the International Union for Conservation of Nature (IUCN), and the swan goose’s global population has been estimated at c.60,000–90,000 (http://www.iucnredlist.org/). In China, the wild swan goose has been designated as a Class II national protected species. Therefore, it’s difficult to obtain wild swan goose samples to carry out genomic studies. Xianghai wetland national nature reserve (44°55′–45°09′N, 122°05′–122°31′E), located in the Jilin province (the northeast region of China), was one of the wild swan goose’s habitats. In 1981, the Xianghai wetland national nature reserve was established, and it is the habitat of many protected migratory birds e.g. *Grus japonensis, Otis tarda*.

We are working at the front line of geese breeding in the Xianghai wetland national nature reserve. Our team consists of researchers from Jilin University and Jiuzhou Flying Goose Husbandry&Technology Co., Ltd., which holds a license to domesticate swan geese in the Xianghai Wetlands. In 1999, their farm was established for domesticating and breeding wild swan geese in Yanya lake in the Xianghai wetland national nature reserve. In recent years, we are working on hybrid goose breeding (swan goose × local domestic goose). We are interested in investigating the genetic difference between the wild swan goose and the domestic goose. Considering that sequencing the wild swan goose provides a valuable resource not only for researchers working on animal conservation but also for those focusing on goose genomic breeding and further genomic investigation. With this in mind, we report and make publicly available whole genome sequencing data for 10 wild swan geese.

## Samples collection and sequencing

The Jiuzhou Flying Goose Husbandry&Technology Co., Ltd. holds a license to domesticate swan geese in the Xianghai Wetlands. The experienced staff randomly picked up wild swan goose eggs from the Xianghai Wetland National Nature Reserve for incubation and rearing. About 2 mL of blood samples were collected from the veins under the wings of the adult swan geese by the experienced staff, and all the swan geese remained healthy after blood collection. Genomic DNA was extracted from the blood following the standard phenol-chloroform extraction procedure. For genome sequencing, at least 0.5 μg of genomic DNA from each sample was used to construct a library with an insert size of 350 bp. Paired-end sequencing libraries were constructed according to the manufacturer’s instructions (Illumina Inc., San Diego, CA, United States) and sequenced on the Illumina HiSeq platform.

## Data quality control and variant calling

The FASTP (Chen et al., 2018) software was used to perform quality control on the raw data. The clean reads were aligned to the swan goose genome (PRJNA826973) using Burrows-Wheeler Alignment Maximal Extract Matches algorithm (Li and Durbin, 2009) with default parameters. The SNPs (Single Nucleotide Polymorphisms) were called using the GATK software (McKenna et al., 2010). For these to be called, the calling quality had to be greater than 20 (base recognition accuracy >99%). The SNPs were filtered with minor allele frequency <0.05 and missing rate >0.10 using the VCFtools (Danecek et al., 2011). The distribution of the SNPs on the chromosomes was plotted using the R “CMplot” package.

## Data description

**FIGURE 1:**
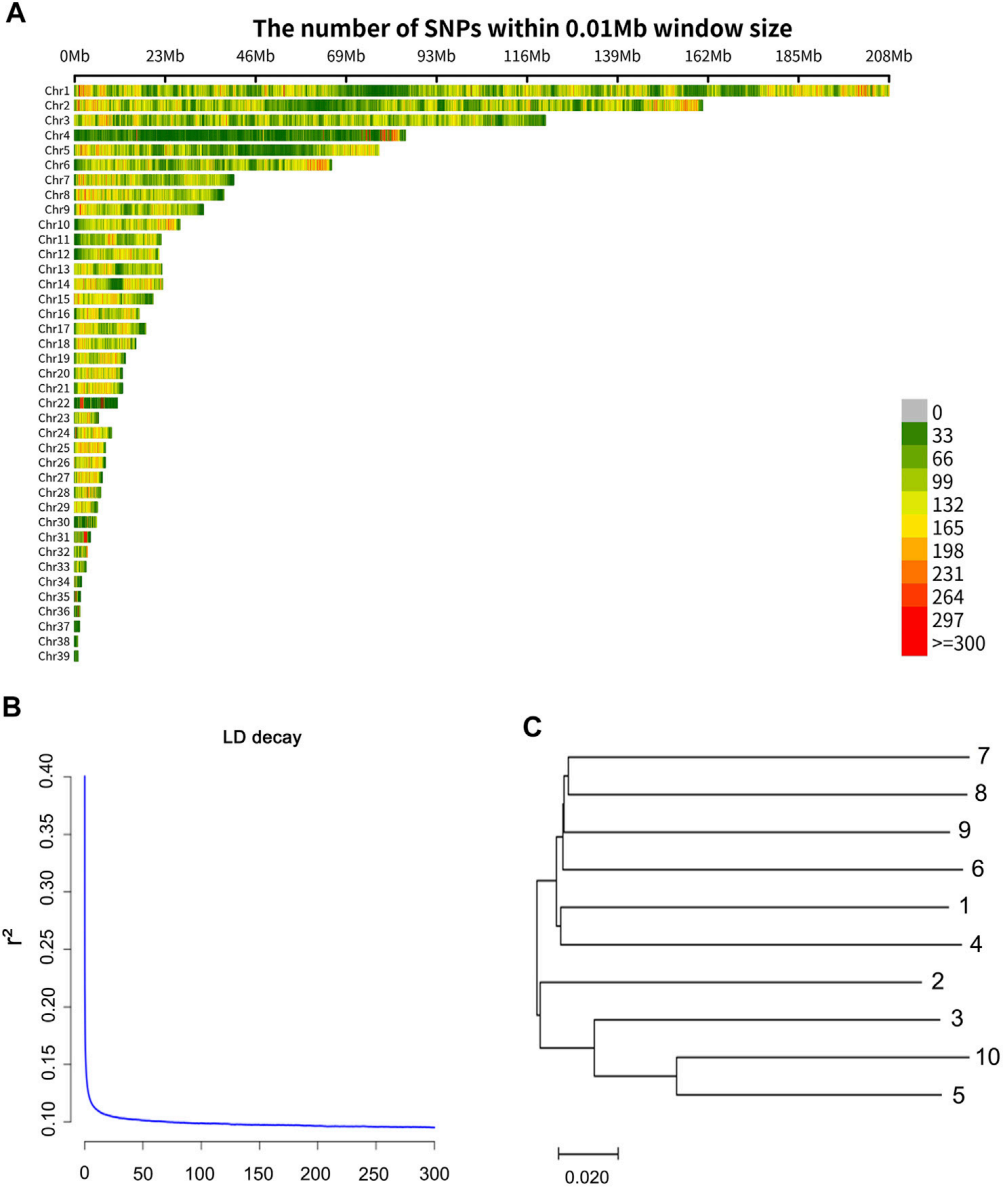
**(A)** Distribution of the SNPs on the chromosomes. The *x*-axis represents the chromosome position (Mb), and the *y*-axis represents the chromosomes. The number of SNPs presented in each 10 kb genome block is displayed by the different colors. **(B)** Extent of LD average r^2^ values at distances up to 300 kb. **(C)** The neighbor-joining tree based on 1-IBS distance.

A total of 162.6 Gbp clean data was obtained. The sequence data were deposited in the NCBI Sequence Read Archive (SRA) and the accession number of the sequencing data is PRJNA814334. One individual was deeply sequenced with 39.6 Gbp data obtained, and the other samples were sequenced with about 13.7 Gbp data obtained. The sequencing information of each sample is shown in Supplementary Table S1. The average mapping rate was 0.90 (mapping quality value ≥20). Finally, a total of 10,727,005 SNPs (minor allele frequency ≥0.05) were obtained, and a total of 7,646,295 (71.28%) transitions (Ts) and 3,080,710 (28.72%) transversions (Tv) were observed. The distribution of the SNPs in 10 kb non-overlapping windows on the chromosomes is provided in Figure 1A. From the information of the SNPs, Linkage disequilibrium (r2) measures were calculated based on the PopLDdecay (Zhang et al., 2019). The decay of LD (linkage disequilibrium) according to distance, for pair-wise SNPs up to 300 kb is shown in Figure 1B. The LD value (r^2^ = 0.2) was about 200 bp in the wild swan goose, and the levels of LD at different distances were presented in Supplementary Table S2.

To provide future researchers with an understanding of the genetic difference between the wild swan goose and the domestic goose, here we compared the swan goose populations with a domestic goose breed, the Zi goose, a local domesticated breed in northeast China. The PCA and LD analysis results are provided in Supplementary Figure S1. The plot of the first two PCA components showed a clear genetic differentiation, along the first component, between the swan geese and Zi geese. In addition, the PCA patterning highlighted a wider genetic differentiation among the swan geese samples than among the Zi geese (Supplementary Figure S1A). Historically, the wild swan geese have not undergone intensive selection as experienced in Zi geese. Hence, it is reasonable to assume that the Swan geese mantain higher levels of genetic variability than Zi geese. This assumption is also supported by the LD analysis. In our study, we find that the LD extend of the Zi geese is higher than the Swan geese (Supplementary Figure S1B). It is known that the LD extent could reflect the history of the population. The strong artificial selection could contribute to increasing the LD extent, although we can't exclude the possibility of other factors such as gene flow.

To provide future researchers with an understanding of the genetic relationship among samples, we constructed an inter-individual neighbor-joining (NJ) tree of the ten swan geese samples (Figure 1C) using the (1-IBS) genetic distances, with the identity-by-state (IBS) values being estimated using PLINK v1.9 (Purcell et al., 2007). Genetic distances between individuals ranged from 0.184 to 0.291, and the average value was 0.272. Moreover, we calculated the inbreeding coefficient of each individual using PLINK v1.9 (Purcell et al., 2007) with the command “het.” The inbreeding coefficient (Supplementary Table S3) ranged from −0.01 to 0.12, and the average value was 0.07. This result indicated that the extent of inbreeding of the wild swan goose population is low.

In conclusion, this paper provides the whole genome resequencing data of ten swan geese, which can serve as a theoretical reference for future exploration of genetic variation or characteristics between swan geese and domestic goose populations, and is of great value in revealing the genetic mechanism of phenotypic differences. For example, by comparing the genome of the wild swan goose to the local goose, researchers may find the clue to the genetic background of the special characteristics of the swan goose, e.g., long-term flying ability.

## Data Availability

The sequence data presented in the study are deposited in the NCBI Sequence Read Achieve (SRA) repository, accession number PRJNA814334. The datasets presented in this study can be found in online repositories. The names of the repository/repositories and accession number(s) can be found in the article/Supplementary Material.
