# Supplementary figures and images for: Whole genome sequencing of a wild swan goose population
Source: Front Genet. 2023 Feb 24;14:1038606. doi: 10.3389/fgene.2023.1038606 (PMC10000724; doi:10.3389/fgene.2023.1038606)

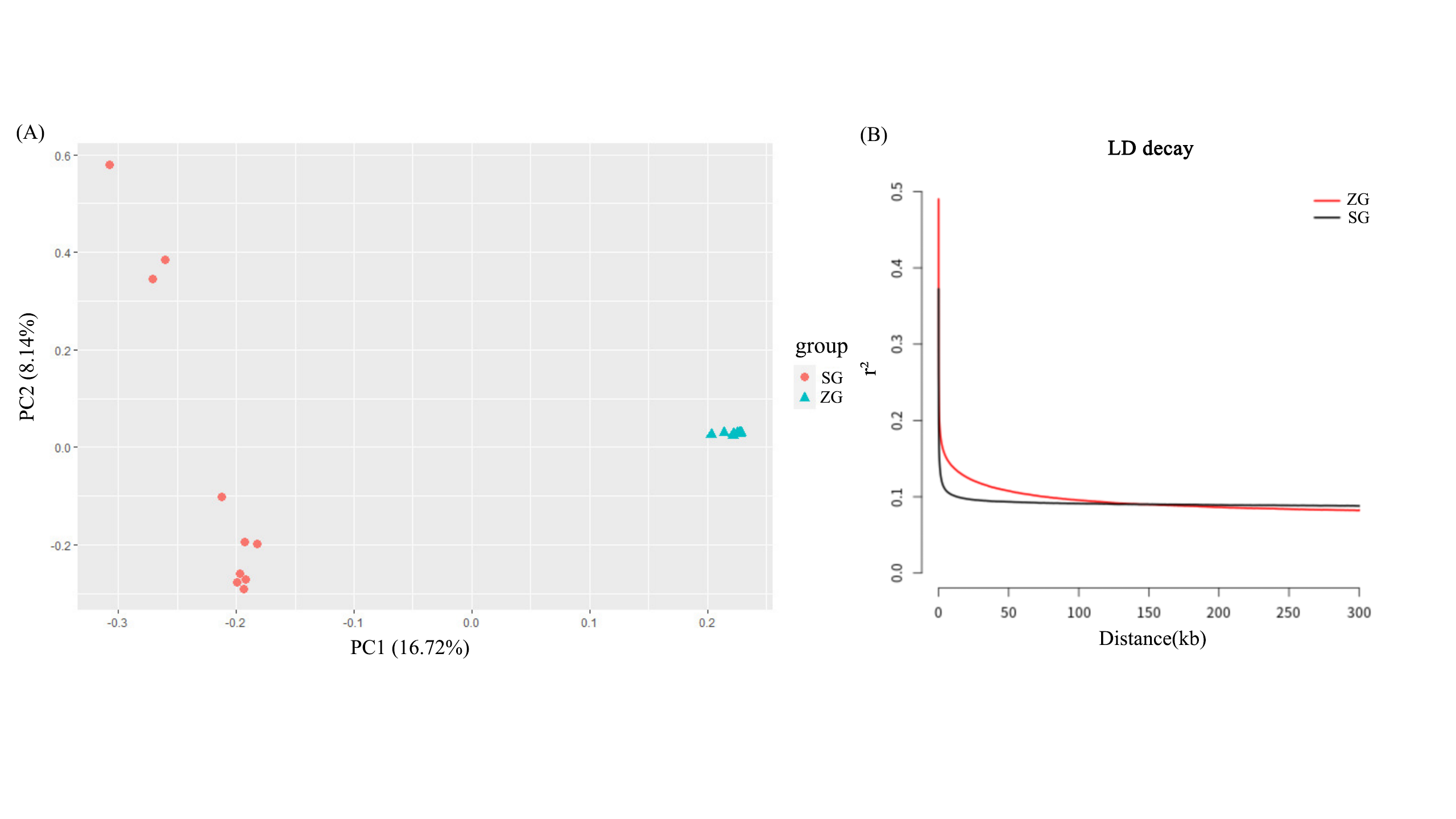

Supplement: Supplementary file 4 [file Image1.PNG]
